# Sequence, Structure, and Functional Space of *Drosophila* De Novo Proteins

**DOI:** 10.1093/gbe/evae176

**Published:** 2024-08-30

**Authors:** Lasse Middendorf, Bharat Ravi Iyengar, Lars A Eicholt

**Affiliations:** Institute for Evolution and Biodiversity, University of Muenster, Huefferstrasse 1, 48149 Muenster, Germany; Institute for Evolution and Biodiversity, University of Muenster, Huefferstrasse 1, 48149 Muenster, Germany; Institute for Evolution and Biodiversity, University of Muenster, Huefferstrasse 1, 48149 Muenster, Germany

**Keywords:** de novo proteins, protein function, structural comparison, protein structure, structure predictions, sequence space

## Abstract

During de novo emergence, new protein coding genes emerge from previously nongenic sequences. The de novo proteins they encode are dissimilar in composition and predicted biochemical properties to conserved proteins. However, functional de novo proteins indeed exist. Both identification of functional de novo proteins and their structural characterization are experimentally laborious. To identify functional and structured de novo proteins in silico, we applied recently developed machine learning based tools and found that most de novo proteins are indeed different from conserved proteins both in their structure and sequence. However, some de novo proteins are predicted to adopt known protein folds, participate in cellular reactions, and to form biomolecular condensates. Apart from broadening our understanding of de novo protein evolution, our study also provides a large set of testable hypotheses for focused experimental studies on structure and function of de novo proteins in *Drosophila*.

**Significance**

De novo proteins emerge from formerly noncoding regions of genomic DNA. While a few de novo proteins have been shown to perform essential functions in different organisms, it is experimentally laborious to discover the structure and the function of de novo proteins, especially at a large scale. To help address this problem, we used machine learning based computational tools to predict the structure and the function of ∼1,500 *Drosophila* de novo proteins, thereby providing a dataset for a focused experimental validation.

## Introduction

Once considered impossible (Zuckerkandl 1975; Jacob 1977), many lines of evidence suggest that functional proteins can emerge from random sequences that have not been subjected to several generations of evolution (Keefe and Szostak 2001; Hecht et al. 2004; Babina et al. 2023). For example, high throughput selection experiments with a large number of random sequences have shown, that some random proteins can mitigate auxotrophy (the inability to metabolize nutrients; Knopp et al. 2021), provide resistance against toxins (Frumkin and Laub 2023), and even catalyze biochemical reactions (Yamauchi et al. 2002; Chao et al. 2013). In accordance with the fact that protein folding is often a critical requirement for protein function, many random proteins have been also shown to have secondary structures (Davidson and Sauer 1994; Davidson et al. 1995; Surdo et al. 2004; Mansy et al. 2007; Tretyachenko et al. 2017). De novo emergence is a phenomenon through which novel protein coding genes arise from nongenic regions of the genome (Tautz and Domazet-Lošo 2011; Carvunis et al. 2012; Schmitz and Bornberg-Bauer 2017; Branden Van Oss and Ruxandra Carvunis 2019; Vakirlis et al. 2020; Bornberg-Bauer et al. 2021). The de novo proteins that emerged have been considered to be the natural equivalent of random sequences, because they emerge from supposedly “random” intergenic regions, and some of their predicted properties such as length, structural disorder and aggregation propensity, resemble that of random proteins, more than that of conserved proteins (Ángyán et al. 2012; Bhave and Tautz 2021; Bornberg-Bauer et al. 2021; Castro and Tautz 2021; Heames et al. 2023; Aubel et al. 2024; Middendorf and Eicholt 2024). For example, de novo proteins in *Drosophila*, are predicted to be more disordered than conserved proteins (Heames et al. 2020; Peng and Zhao 2024; Middendorf and Eicholt 2024), which can be partially explained due to higher GC content of the former (Landry et al. 2015; Zheng and Zhao 2022). While the structure of large sets of de novo proteins have been computationally analyzed (Basile et al. 2017; Schmitz et al. 2018; Heames et al. 2020; Vakirlis et al. 2020b; Chen et al. 2024; Peng and Zhao 2024), the structures of only four de novo proteins have been experimentally approximated (Bungard et al. 2017; Baalsrud et al. 2018; Lange et al. 2021; Matsuo et al. 2021). Determining the function of de novo genes and proteins is another challenging task. It involves identifying the cell types and stages in which de novo proteins may be involved and testing their phenotypic effects using genetic tools (Chen et al. 2010; Reinhardt et al. 2013; Gubala et al. 2017; Lange et al. 2021). Nonetheless, functional de novo proteins indeed exist and have been identified in organisms as diverse as insects (*D. melanogaster* among others), plants (*Arabidopsis thaliana*), fungi (*Saccharomyces cerevisae*), arctic codfish *(Boreogadus saida)*, mice (*Mus musculus*), and humans (*Homo sapiens*) (Begun et al. 2007; Cai et al. 2008; Heinen et al. 2009; Li et al. 2009; Chen et al. 2010; Li et al. 2010; Reinhardt et al. 2013; Li Yan et al. 2014; McLysaght and Guerzoni 2015; Gubala et al. 2017; Klasberg et al. 2018; Xie et al. 2019; Zhuang et al. 2019; Lange et al. 2021; Matsuo et al. 2021; Rivard et al. 2021; Vakirlis et al. 2022; Linnenbrink et al. 2024). Experimental structure determination is a laborious process that cannot be performed in a high throughput manner. This is especially difficult for de novo proteins because of high aggregation propensity and low solubility in vitro (Eicholt et al. 2022). Despite the increasing numbers of solved structures, novel structures, whether they be folds or domains, were rarely ever found (Grant et al. 2004; Levitt 2009; Tóth-Petróczy and Tawfik 2014). However, the recent advancements in high-throughput structure predictions through computational techniques, have led to discovery of novel folds (Durairaj et al. 2023). Since de novo proteins are void of ancestry from conserved protein families, they could provide rare structural novelty (Bornberg-Bauer et al. 2021). From another perspective, the occurrence of conserved or ancient structural folds in de novo proteins could suggest a high level of evolutionary accessibility in sequence space. This might explain the emergence of these folds during the early stages of protein evolution (Lupas et al. 2001; Alva et al. 2010; Kopec and Lupas 2013; Alva et al. 2015; Romero Romero et al. 2016). A protein’s structure can provide some clues about its function (Orengo et al. 1999). For example, one can reasonably guess the function of an uncharacterized protein by comparing its structure to that of a known functional protein (Nomburg et al. 2024). Although, protein function is often attributed to its structure, and unfolded proteins were assumed to be toxic, many studies show that disordered proteins can be functional (Ali and Ivarsson 2018; Jemth et al. 2018; Deiana et al. 2019). For example, disordered proteins can help form intracellular condensates (or membraneless organelles) that have been shown to play a major role in the cellular physiology of diverse organisms (Hyman et al. 2014; Lin et al. 2017). Because de novo proteins could be a source of novelty, with regards to both structure and function, we aimed to understand their structures and possible functions through computational analyses. To this end, we studied a previously characterized set of 2,510 putative de novo proteins from the *Drosophila* clade (Heames et al. 2020; Middendorf and Eicholt 2024). We used a multifaceted approach to analyze these de novo proteins. First, we used Foldseek (van Kempen et al. 2024) to find experimentally known protein structures (Protein Data Bank, Berman et al. 2000) and predicted protein structures (AlphaFold database, Varadi et al. 2021) that are similar to the AlphaFold2 (AF2) (Jumper et al. 2021) predicted structures of our de novo proteins. Second, we predicted the functions of our de novo proteins using DeepFRI (Gligorijević et al. 2021), a machine learning-based tool that predicts functional annotations (gene ontology terms) using protein structure and sequence features. Because many of our de novo proteins were predicted to be disordered de novo proteins, we hypothesized that they could form biomolecular condensates (Uversky 2017). To test this hypothesis, we predicted the condensate forming propensity of our de novo proteins using PICNIC (Hadarovich et al. 2023), an algorithm that is based on predicted structure (AF2), predicted disorder (IUPred2A), as well as sequence complexity. Understanding the condensate forming behavior of de novo proteins could elucidate their potential involvement in the formation of membraneless organelles, offering an evolutionarily and biophysically feasible mechanism for their integration with the cellular physiology. Finally, we mapped the de novo proteins on the protein sequence space in relation to random and conserved proteins. To this end, we used protein language model ESM2-650 M, that can predict several biophysical features from sequences, embedding their abstracted properties in the form of numerical values (Lin et al. 2023). Our method allowed us to map different sequences with better resolution than by the analyses of individual properties separately (Agozzino and Dill 2018; Weidmann et al. 2019; Heames et al. 2023; Aubel et al. 2024). With these multifaceted analyses we found that some de novo proteins can indeed adopt structures similar to known proteins and can have possible cellular activities including localization to specific organelles. We also found that some de novo proteins are likely to form biomolecular condensates. However, with our language model analysis we found that the majority of de novo proteins look distinct from conserved proteins of similar length and resemble more the random proteins. Overall, our work enhances our understanding of how de novo proteins can not only develop features already known to the living systems but can also be a source for evolutionary novelty.

## Results

### A Few De Novo Proteins can Indeed Adopt Known Structures

To understand if de novo proteins can form known protein structures, we compared their predicted structure to that of conserved proteins. Recent studies have shown that structure predictions are not very reliable for de novo proteins (Aubel et al. 2023; Liu et al. 2023; Middendorf and Eicholt 2024), and that many predicted structures are also thermodynamically unstable (Peng and Zhao 2024).

**Fig. 1.**
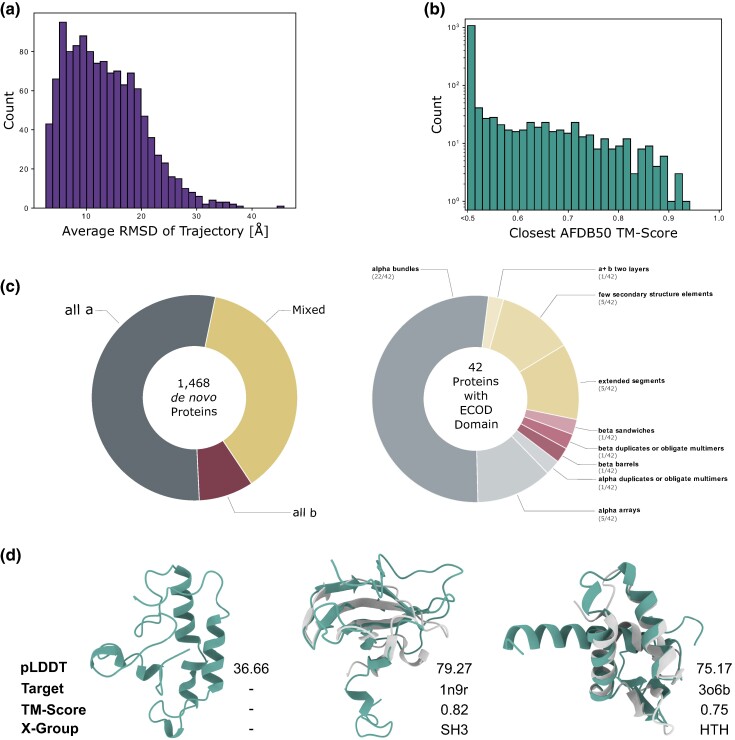
Structural diversity of de novo evolved proteins. a) Distribution of the average RMSD (*X* axis) per MD simulation trajectory. Average RMSD of MD simulation triplicates per de novo protein, only for proteins with (i) less than 30% disorder predicted by flDPnn and (ii) less than 95% of their residues annotated as α-helices via DSSP (1,468 of 2,510 proteins). b) Distribution of the TM-score (*X* axis) for the mapping of de novo proteins (MD-refined structures) to the most similar protein structure in AFDB50, excluding proteins from *Drosophila*. TM-scores below 0.5 indicate no similarity to any protein structure in AFDB50. c) Structural classification of de novo proteins. We assigned a structural class to each of the 1,468 de novo proteins based on the DSSP annotations of their MD-refined structures (inner circle). To identify annotated protein domains in de novo proteins, we aligned their MD refined structures to structures in the PDB. We assigned each de novo protein with the ECOD domain of its highest scoring hit from the PDB, given the TM-score was greater than 0.5 and the alignment covered at least 80% of the PDB target. We assigned the 42 de novo proteins, that qualified the above criteria, with an ECOD domain from multiple domain architectures (outer circle). d) Examples of de novo proteins without structural similarity to proteins in the AlphaFold database (Droso_1087), or with similar structure to an ECOD X-group (Droso_1446 and Droso_4840; aligned with their closest hit in the PDB).

Therefore, we refined the AF2 predicted structures of *Drosophila* de novo proteins from our previous study (Middendorf and Eicholt 2024) by performing molecular dynamics simulations for 100 ns in triplicates, for each de novo protein. We thus refined the predicted structures of 1,468 de novo proteins with <30% disorder according to flDPnn and <95%α-helices annotated by DSSP. Our MD simulations suggest that most de novo proteins exhibit structural flexibility, as indicated by the large root mean square deviation (RMSD) values (Fig. 1a and supplementary Fig. S3, Supplementary Material online) (Bornot et al. 2011; Hrabe et al. 2016). Next, we searched for conserved proteins that have predicted structures similar to those of de novo proteins, using Foldseek (van Kempen et al. 2024). Specifically, with MD refined structures as queries, and the AFDB50 (Varadi et al. 2021) as the target, we observed that the majority of de novo proteins did not have a significant structural similarity to the conserved proteins in AFDB50 (TM score <0.5, Fig. 1b). This was also the case for AF2 predicted structures of de novo and random proteins without MD simulations (supplementary Fig. S1, Supplementary Material online and supplementary Fig. S2, Supplementary Material online). We hypothesized that structurally stable/rigid (small RMSD) proteins may have more similar structures, than structurally unstable/flexible proteins. However, we did not find any significant correlation (Pearson ρ−0.31, $P=1.82\times 10^{-29}$) between RMSD of de novo proteins during MD simulation and their similarity (TM score) to protein structures in AFDB50 (supplementary Fig. S4, Supplementary Material online). In general, our observations of only rare structural similarities are in line with the de novo status of our proteins, aligning with the notion that structure is more conserved than sequence (Illergård et al. 2009). To investigate whether these de novo proteins can adopt known structures, we performed a structural mapping of de novo proteins with experimentally validated structures in the Protein Data Bank (PDB) (Berman et al. 2000), using Foldseek. We then extracted the ECOD domain (Cheng et al. 2014) annotations for matches found in the PDB. Out of the 1,468 de novo proteins analyzed, 42 showed structural alignment with proteins having an architecture annotation in ECOD (Fig. 1c). Ten of these 42 de novo protein sequences could also be assigned to sequences in ECOD using hmmscan (Johnson et al. 2010), four of those ten to the same ECOD domains as found using Foldseek (Droso_1511, 4476, 536, 5684; supporting information). No hits were found using hmmscan against Pfam (Mistry et al. 2021). Prior to MD simulation, 119 predicted structures could be mapped to PDB structures (supplementary Fig. S1, Supplementary Material online). Figure 1d presents three examples of these findings consisting of a structurally unalignable de novo protein, one similar to an SH3 fold, and another resembling an HTH fold. Both SH3 and HTH folds are considered highly conserved and ancient folds (Rosinski and Atchley 1999; Grishin 2000; Kishan and Agrawal 2005; Alvarez-Carreño et al. 2021). These three example proteins have emerged less than 5 million years ago (mya) (Heames et al. 2020). This was also the case for 35 out of 42 de novo proteins structurally aligned to ECOD architectures (supplementary Fig. S3, Supplementary Material online). However, young de novo proteins did not align more frequently to ECOD architectures than expected by their frequency in our dataset (Pearson’s $\chi^{2}$-test; P=0.99). Overall, our structure search analysis shows that while most de novo proteins are likely to have novel or uncommon structures, a minority of them, including young de novo proteins, can indeed adopt well known protein structures, commonly consisting of α-bundles, arrays or a composite of secondary elements (Fig. 1c).

### Some De Novo Proteins May Be Involved in Biochemical Reactions and Cellular Components

**Fig. 2.**
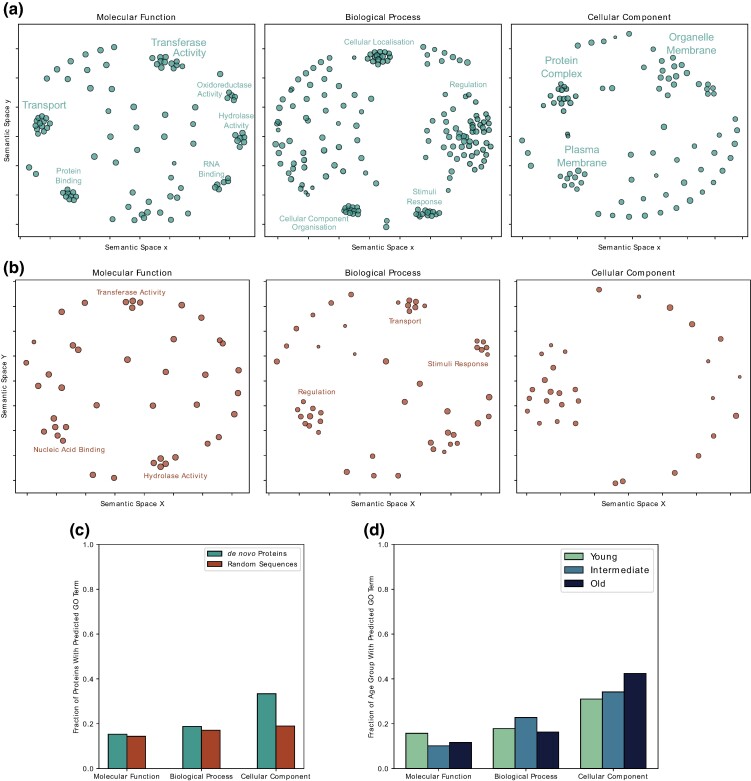
GO terms of random and de novo proteins predicted with DeepFRI We predicted GO terms of de novo proteins (a) and random sequences (b) with DeepFRI and clustered them based on semantic similarity with REVIGO. We visually identified GO term clusters manually annotated with a generic term that describes all the GO terms within the respective cluster. c) Fraction of de novo and random proteins (vertical axis) predicted with a GO term per GO term category (horizontal axis). Differences observed in the frequency of confident annotations with GO terms between random and de novo proteins were only significant for the “Cellular Component” GO term class (Fisher’s Exact Test; $P=7.5\times 10^{-23}$). d) Fraction of de novo proteins in different age groups (vertical axis) with a predicted GO term (horizontal axis). Old de novo proteins were significantly more often annotated with a GO term in the *“Cellular Component”* category than expected by chance (Pearson’s $\chi^{2}$-test; P<0.05).

Information on biological activities and functions, is available for only a handful of de novo proteins (Bornberg-Bauer et al. 2021; Weisman 2022). Even though a nonfunctional protein can be fixed in a species and can persist in the genome for many generations (Gould and Lewontin 1979; Stoltzfus 1999; Lynch et al. 2016), the existence or gain of a biological activity could accelerate the evolutionary fixation of de novo proteins. Thus functional annotation of de novo proteins could help understanding their evolutionary history and significance. However, their lack of homology, makes their functional annotation challenging. To overcome this problem, we used DeepFRI to functionally annotate de novo proteins with gene ontology (GO) terms. Unlike homology based techniques, DeepFRI combines a protein language model, trained on the sequences of Pfam domains (Mistry et al. 2021), and a graph convolutional network that represents amino acid interactions derived from protein structure (Gligorijević et al. 2021). DeepFRI is also trained on the GO terms associated with different structures. We did not filter protein sequences according to any structural criteria, because DeepFRI can de-noise predicted protein structures (Gligorijević et al. 2021). For the functional annotation using DeepFRI, we used AF2 predicted structures of randomly generated proteins, and the MD refined structures of de novo proteins. We summarized and clustered the predicted GO terms based on their semantic similarity, and projected them in a 2D semantic space using REVIGO (Supek et al. 2011) (Fig. 2a and b). We identified these GO term clusters visually and manually annotated them based on the GO terms within the cluster. We performed this analysis for both de novo and random proteins. With our analysis, we found that a small fraction of de novo and random proteins could be confidently annotated with GO terms for all the three GO classes (“Molecular Function,” “Biological Process,” and “Cellular Component”; Fig. 2c). The GO term class “Cellular Component” had the highest fraction of confident predictions with ≈33% and ≈19% predictions for de novo and random proteins, respectively. Furthermore, de novo proteins were significantly more often annotated under “Cellular Component” than random proteins (Fisher’s exact test; $P=7.5\times 10^{-23}$). We observed no significant differences in the other two GO classes between de novo and random sequences. Within the “Cellular Component” class we found that de novo proteins were frequently annotated with the GO terms pertaining to their localization in protein complexes or membranes. This finding is in agreement with existing studies on de novo proteins in yeast and rice (Vakirlis et al. 2020b; Chen et al. 2024). In contrast, we could not find random proteins to be enriched in any specific GO terms cluster within the “Cellular Component” class (Fig. 2). This suggests that they can localize to many different cellular compartments. Specifically, we found that some random proteins can possibly localize to the following compartments: nucleus (GO:0005634), mitochondrion (GO:0005739), vesicles (GO:0031982), and membranes (GO:0016020).

Both de novo proteins and random sequences show a broad variety of GO terms in the other two GO classes with only a few prominent clusters within the semantic space (Fig. 2a and b). Interestingly, de novo proteins and random sequences appear to have similar “Molecular Function” and to be involved in similar categories of “Biological Process”. Regarding their “Molecular Function”, they both were associated with GO terms pertaining to “hydrolase activity” and “transferase activity.” The “Biological Process” in which both de novo and random sequences were predicted to participate are “stimuli response” and “regulation.” Next, we analyzed the impact of evolutionary age on functional annotation of de novo proteins using GO terms. Specifically, we catergorized them as old (<5 mya), intermediate (5–30 mya), and young (>30 mya), based on previous studies (Heames et al. 2020; Middendorf and Eicholt 2024). Because young de novo proteins were more frequent than the older proteins in the dataset, we normalized the number of proteins with predicted GO terms to the number of proteins in the respective age group. Only for the GO term category “Cellular Component”, old de novo proteins were annotated more frequently than expected by chance (Pearson’s $\chi^{2}$-test; P<0.05). We also specifically analyzed the GO terms for the 42 de novo proteins with a good structural similarity to ECOD domains. We found that six are predicted with GO terms in “Molecular Function”, 11 with GO terms in “Biological Process” and 16 with GO terms in “Cellular Component” (Supplementary material online).

### Subset of De Novo Proteins may Form Biomolecular Condensates

**Fig. 3.**
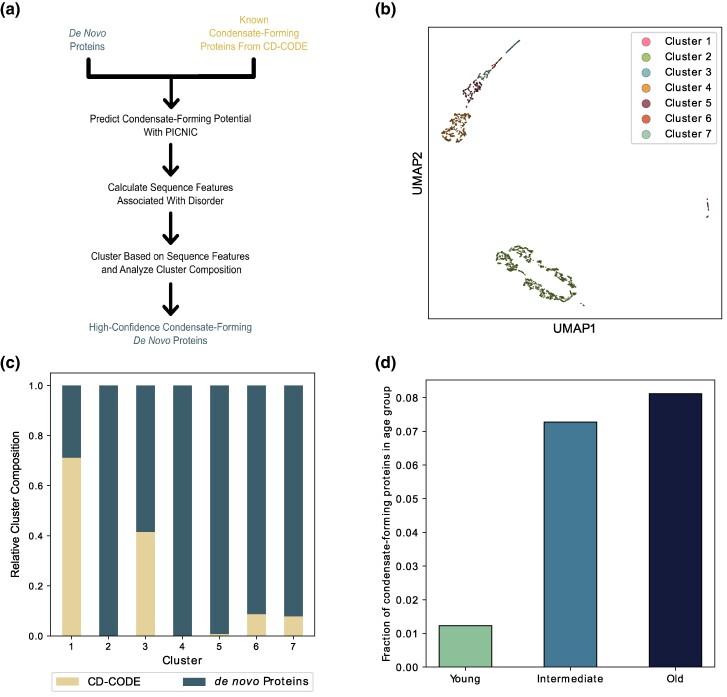
Identification of condensate-forming de novo proteins. a) Workflow for the identification of condensate-forming de novo proteins. We predicted condensate-forming potential of de novo proteins and known condensate-forming proteins from the CD-CODE database with PICNIC. For both groups of proteins, we calculated the sequence features associated with the functions of intrinsically disordered regions were calculated. Subsequently, we clustered all proteins based on these sequence features using hdbscan, and the analyzed the clusters for their constituent proteins. b) Clusters of de novo proteins and known condensate-forming proteins based on sequence features associated with the function of intrinsically disordered proteins. c) Constitution of the identified clusters based on protein type. We classified the 63 de novo proteins from clusters 1 and 3 were as high-confidence condensate-forming proteins. d) Fraction of de novo proteins from the respective age groups that were classified as high-confidence condensate-forming proteins. The age groups *Intermediate* and *Old* contained significantly more high-confidence condensate-forming proteins than expected by chance (Pearson’s $\chi^{2}$-test; $P<5\times 10^{-54}$).

Biomolecular condensates are membraneless compartments formed by proteins via liquid–liquid phase separation and are involved in several processes such as stress response and regulation of transcription (Hyman et al. 2014; Tsang et al. 2020). We observed that GO terms pertaining to RNA binding, transferase activity, and hydrolase activity that were predicted for de novo proteins (Fig. 2), are also important features of condensate-forming proteins (Hadarovich et al. 2023). Therefore, we predicted the propensity of de novo proteins for condensate-formation. To this end, we used the complete set of de novo proteins from the previous studies (Heames et al. 2020; Middendorf and Eicholt 2024) and not only the de novo proteins for which we performed MD simulations. We did so because we only performed MD simulations for de novo proteins with a predicted disorder ≤30%, but disorder is considered an important feature of condensate-forming proteins (Uversky 2017; Boeynaems et al. 2018). To investigate the condensate-forming potential of de novo proteins, we used the computational tool PICNIC (Hadarovich et al. 2023) that in turn uses AF2 predicted structures and disorder prediction tool IUPred2A, to predict condensate formation propensity. It has been shown, that both AF2 and IUPred can make qualitatively discordant predictions of de novo proteins (Aubel et al. 2023; Middendorf and Eicholt 2024). Therefore, we performed additional analyses to ensure a high-confidence prediction of condensate-forming de novo proteins (Fig. 3a). Specifically, we retrieved 175 known condensate-forming conserved proteins from the CD-CODE database (Rostam et al. 2023) and used them as a positive control dataset. For all these proteins, we calculated the sequence features that are associated with the biological function of their intrinsically disordered regions, e.g. amino acid homorepeats, sequence complexity, and net charge (Zarin et al. 2021). We clustered the sequences based on these features using HDBSCAN (McInnes et al. 2017) after reducing their dimensionality from 131 to 5 using Uniform Manifold Approximation and Projection (UMAP) (McInnes et al. 2018). UMAP is a commonly used nonlinear dimensionality reduction tool (in contrast to principal component analysis, which is linear). We further used UMAP to project the sequences based on their calculated sequence features in a 2D space solely for visualization purposes and marked the different clusters identified with HDBSCAN (Fig. 3b). We identified seven clusters of different sizes. Of these, cluster 1 and cluster 3 contained most proteins (88.6%) of the CD-CODE database that we used in our analysis (Fig. 3c). The de novo proteins in cluster 1 and cluster 3 with a PICNIC score greater than 0.5 can be considered high-confidence condensate forming proteins, because they are not only predicted by PICNIC according to its own criteria, but they also have a similar sequence composition as experimentally validated condensate-forming proteins. In total, we identified 63 such high-confidence condensate-forming de novo proteins. As UMAP dimensionality reduction is affected by the choice of hyperparameters (Wang et al. 2021; Marx 2024), we investigated the robustness of our results with different UMAP hyperparameters prior to clustering with HDBSCAN. We find that the majority of the 63 high confidence condensate forming de novo proteins we identified (using 15 n_neighbors) are also identified using different values of UMAP hyperparameters (supplementary Fig. S6, Supplementary Material online). We next analyzed the age groups of these condensate forming de novo proteins. When normalized by the number of proteins per age group, we found intermediate and old de novo proteins to be 5.9- and 6.6-fold more often predicted to form condensates than young de novo proteins, respectively (Fig. 3d). Furthermore, intermediate and old de novo proteins contained significantly more high-confidence condensate-forming proteins than expected by chance (Pearson’s $\chi^{2}$-test; $P<5\times 10^{-54}$). We further found that three de novo proteins predicted to form condensates, are also annotated as α-bundles (Droso_3599, Droso_4131, & Droso_5917), and two of them (Droso_148 & Droso_4483) were annotated with the GO term “RNA binding.”

### Protein Language Models Show That De Novo and Conserved Proteins Occupy Distinct Regions of the Sequence Space

**Fig. 4.**
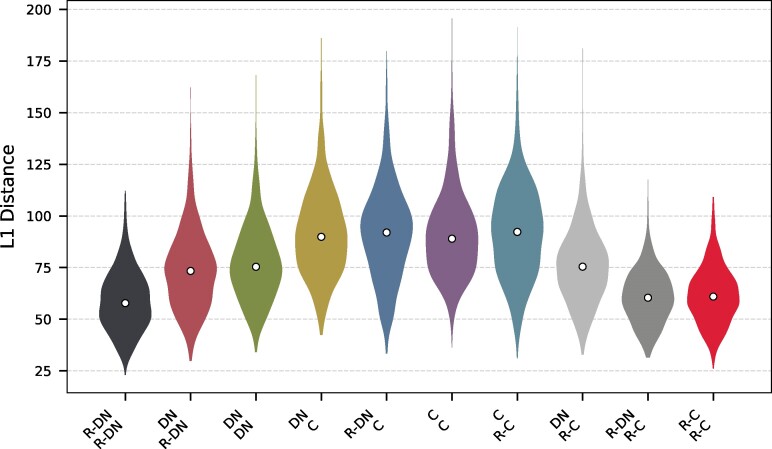
Distribution of pairwise L1 distances between the four different datasets of de novo and conserved sequences and their respective randomized sets. We used the protein language model ESM2-650 M to generate a numerical representation of the de novo (DN), conserved proteins (C) and randomized sequences of both (R-DN and R-C). Based on the embeddings of ESM2-650 M, we calculated the pairwise L1 distance between the four datasets and within. Accordingly, de novo proteins (DN) resemble more random proteins (DN-R & C-R), of a similar length distribution, than conserved proteins (C), independent of the amino acid distribution. DN-R is the randomized set based on de novo sequences and C-R of conserved sequences.

Although we found that some de novo proteins may be structurally similar to known proteins, we do not yet know if evolutionary origin indeed determines the structural properties of a protein. Indeed, many studies have compared a handful of features such as structural disorder, protein composition, and aggregation propensity between de novo and conserved proteins (Knowles and McLysaght 2009; Ekman and Elofsson 2010; Landry et al. 2015; Wilson et al. 2017; Klasberg et al. 2018; Schmitz et al. 2018; Vakirlis et al. 2018; Heames et al. 2020, 2023; Peng and Zhao 2024; Middendorf and Eicholt 2024). However, these analyses may not provide reliable inferences because they use tools depending on limited data (e.g. TANGO/IUPred) (Fernandez-Escamilla et al. 2004; Erdős et al. 2021), and because the different features are analyzed in isolation. Language models use machine learning to analyze several hidden parameters (and their interactions) simultaneously using sequence information alone. Indeed, protein language models have proved extremely adept at predicting and designing protein structures (Alley et al. 2019; Heinzinger et al. 2019; Chowdhury et al. 2022; Ferruz and Höcker 2022; Ferruz et al. 2023; Lin et al. 2023; Madani et al. 2023). Therefore, we used the ESM2 protein language model to compare four different kinds of protein sequences in our dataset. As before, we analyzed de novo proteins (DN) and their randomized counterpart based on the same amino acid and length distribution (R-DN). Additionally, we included conserved sequences from the *Drosophila* clade (C), matched to the de novo protein dataset length distribution, and a corresponding randomized sequence set to these conserved proteins (R-C). Specifically, we generated a numerical vector for each protein sequence using the ESM2 language model with 650 million parameters (ESM2-650 M) (Lin et al. 2023). Each vector contains 1280 elements, that denote an abstraction of different sequence features predicted by the model. To quantify how distant our sequences are in sequence space according to the embeddings of ESM2-650 M, we calculated the Manhattan distance (or L1 norm) between every pair of protein numerical sequences, a method particularly effective for multidimensional data with potential extreme outliers (Barrodale 1968). Our findings indicate that the distances between de novo and conserved proteins are generally larger than those between sequences within each of these categories (one-sided Mann–Whitney *U* test; $P<10^{-15}$; Fig. 4). We also found that the distances between the de novo and conserved proteins are generally larger than the distances between the de novo and the random proteins (one-sided Mann–Whitney *U* test; $P<10^{-15}$; Fig. 4). Comparing the median L1 distance of all de novo proteins to conserved sequences (91.13) with the median distance of the 42 structurally annotated de novo proteins to their nearest conserved neighbor (42.94), we find those 42 to be significantly closer in sequence space (one-sided Mann–Whitney *U* test; $P<1.44^{-17}$; supporting information). We found that de novo proteins were closer to conserved-randomized proteins than to conserved proteins (one-sided Mann–Whitney *U* test; $P<10^{-99}$; Fig. 4). Overall our analyses suggest that despite certain structural similarities, de novo proteins are, distinct from conserved proteins at the sequence level, and bear a closer resemblance to random sequences.

## Discussion

Most proteins can be grouped into families based on their sequence similarity, evolutionary ancestry, structural folds, and biochemical functions (Chothia 1992). De novo proteins are exceptions as they do not belong to any established protein family, because they not only originate from nongenic DNA sequences (lack of ancestry) but also lack sequence and structural similarity to other proteins (Schlötterer 2015; Bornberg-Bauer et al. 2021). This makes it challenging to annotate functions to de novo proteins based on our knowledge of conserved proteins. Despite their dissimilarity with known proteins, de novo proteins have been shown to perform biological functions and improve the survival and fitness of the organisms that express them (McLysaght and Guerzoni 2015; Cai et al. 2008; Heinen et al. 2009; Li et al. 2009; Chen et al. 2010; Li et al. 2010; Reinhardt et al. 2013; Li Yan et al. 2014; Gubala et al. 2017; Xie et al. 2019; Zhuang et al. 2019; Lange et al. 2021). Advanced computational methods using deep learning have been able to solve problems at an unprecedented scale. For example, AF2 resulted in an exponential increase in the number of computationally predicted protein structures (Varadi et al. 2021) Therefore, we applied some of these deep learning based tools to elucidate the possible structure and function of de novo proteins.

First, we searched for conserved proteins that may be structurally similar to de novo proteins using Foldseek. Most de novo proteins did not bear a significant resemblance to known protein structures, in accordance with their nongenic evolutionary origin, and distinctiveness of their sequence and biophysical properties as shown by previous studies (Heames et al. 2023; Aubel et al. 2024). This lack of resemblance could exist because de novo proteins are highly disordered (Middendorf and Eicholt 2024; Peng and Zhao 2024), and can contain rare secondary structures like $3_{10^{-}}$ or *π*-helices (Chen et al. 2024), that could make structural alignment complicated.

While we attempted to refine AF2 predicted structures of de novo proteins through molecular dynamics (MD) simulations, it is important to note that many de novo proteins may reside in nonaqueous environments such as cell membranes (Fig. 2) (Vakirlis et al. 2020b), may only fold upon interaction with other proteins (Chen et al. 2024) and may be part of multimers (Lynch 2012; Jayaraman et al. 2022; Schulz et al. 2022; Malik et al. 2024). We did not consider all these possibilities in our MD simulations due to computational limitations. Nonetheless, the majority of individual de novo proteins were predicted to be disordered or, if structured, to predominantly form simple α-helices (Heames et al. 2023; Peng and Zhao 2024; Aubel et al. 2024; Middendorf and Eicholt 2024), a trend attributed to many de novo proteins being too short to form globular structures (Shen et al. 2005; Aubel et al. 2024). Our current study corroborates these observations. The frequent emergence of single α-helices in de novo proteins can be attributed to the lower stereochemical and thermodynamical requirements of α-helices (Barlow and Thornton 1988; Greenwald and Riek 2012). This is further underlined by 27/42 ECOD architectures found in our de novo protein dataset being either alpha bundles or alpha arrays. Additionally, most de novo proteins annotated with an ECOD architecture belong to the youngest age group (>5 mya), consistent with their higher frequency in the dataset (supplementary Fig. S5, Supplementary Material online), highlighting that de novo proteins can indeed emerge well-folded (Bornberg-Bauer et al. 2021; Peng and Zhao 2024). On rare occasions where de novo proteins exhibit structural configurations beyond single α-helices, they can resemble common and ancient folds such as SH3 or HTH (Fig. 1d). This observation implies that these widespread evolutionary folds, which are evolutionary successful and easily tolerated by cells, are more accessible in sequence space (Taverna and Goldstein 2000; Shakhnovich et al. 2005; Goldstein 2008), even for sequences that have not been shaped by millions of generations of evolution. Despite identifying some de novo proteins with structural similarity to existing structures, we did not find any novel folds among our candidate proteins, unlike other studies that investigated a much larger set of proteins (Durairaj et al. 2023) (Fig. 1b and d). Our finding that only 10 out of 42 sequences align to ECOD domains via hmmscan, with only four of these aligning to the same F-group, further highlights that de novo proteins can exhibit similarity to structural domains despite lacking sequence similarity. By employing the deep learning based functional annotation tool, DeepFRI (Gligorijević et al. 2021), we found that de novo proteins are associated with a wide array of Gene Ontology (GO) terms, spanning all three GO categories, with several distinct clusters emerging within the semantic field. We show that de novo and random proteins have a similar frequency of confident annotations with GO terms from the “Molecular Function” and “Biological Process” class. However, de novo proteins were significantly more often annotated with GO terms of the “Cellular Component” class than the random proteins, even though the former have a relatively small evolutionary age (Fig. 2c). This suggests that young de novo proteins can rapidly acquire features that enable them to be targeted to specific cellular compartments. Their involvement in a range of “Molecular Function” (like hydrolase activity and transferase activity) and “Biological Process” (such as stimuli response, regulation, and cellular location) underscores their potential impact on the cellular physiology. Interestingly, the similarity in “Molecular Function” and involvement in “Biological Process” between de novo proteins and random sequences could imply a certain level of functional redundancy in the sequence space. This observation might suggest that the emergence of function from novel proteins, even though random sequences, could be a more probable phenomena than previously thought. Finally we emphasize that, while efforts to deduce protein function based on structural similarity is ongoing (Gligorijević et al. 2021; Nomburg et al. 2024), numerous instances exist where proteins with similar structures perform different functions, and vice versa (Finkelstein et al. 1993; Govindarajan and Goldstein 1996; Galperin et al. 1998; Martin et al. 1998).

The association of de novo proteins with biophysical reactions such as RNA binding, and biochemical reactions similar to transferases, and hydrolases, presents an intriguing avenue for understanding their functional capacities and evolutionary significance. This is especially interesting because RNA binding and hydrolase-activity are thought to be conferred even by primordial folds (Vyas et al. 2021; Longo et al. 2022; Seal et al. 2022; Weil-Ktorza et al. 2023) and could possibly been important during origin of life. Both these molecular activities, and a highly disordered structure, are also exhibited by condensate-forming proteins (Hadarovich et al. 2023). Therefore, we investigated the possibility of de novo proteins to be involved in formation of biomolecular condensates. Biomolecular condensates, formed through liquid–liquid phase separation by proteins, are critical in various biological processes and such a propensity exists even for proteins with ancient and simple folds (Longo et al. 2020). The use of PICNIC (Hadarovich et al. 2023) to predict the involvement of de novo proteins in biomolecular condensates represents an innovative approach, albeit with limitations. The reliance on AF2 predictions and IUPred2A as input requirements, introduces a degree of uncertainty, especially given the discordant predictions of these tools between de novo and conserved proteins (Middendorf and Eicholt 2024). This necessitated further analysis to establish a high-confidence set of condensate-forming de novo proteins, leveraging the CD-CODE database (Rostam et al. 2023) as a reference.

The identification of clusters based on sequence features associated with intrinsically disordered regions of proteins is particularly noteworthy. The fact that clusters 1 and 3, which have a high fraction of members from the CD-CODE database, include ≈12% of all de novo proteins with a PICNIC score greater than 0.5, is compelling. It suggests that these de novo proteins not only have the potential to form condensates but also share sequence composition with experimentally validated condensate-forming proteins. The discovery of 63 high-confidence condensate-forming de novo proteins contributes to our understanding of the functional diversity of these proteins. This finding expands the realm of de novo protein functionality beyond traditional views, indicating their potential involvement in complex cellular mechanisms like phase separation. Considering that phase separation is involved in spermatogenesis (Parvinen 2005; Kang et al. 2022), and that de novo proteins show biased expression in testis (Levine et al. 2006; Neme and Tautz 2013; Palmieri et al. 2014; Zhao et al. 2014; Kondo et al. 2017; Nyberg and Carthew 2017; Heames et al. 2020; Peng and Zhao 2024), being involved in biomolecular condensates suggests a possible mechanism by which de novo proteins could play a role in spermatogenesis (Gubala et al. 2017; Lange et al. 2021; Rivard et al. 2021). Moreover, our analysis of the age groups of these de novo proteins revealed that intermediate and old de novo proteins are significantly more likely to form condensates than their younger counterparts. This observation is intriguing as it could imply two scenarios. First, as de novo proteins evolve and mature, they acquire and refine their ability to participate in cellular processes like biomolecular condensation and thereby their function. Under this scenario, the de novo proteins could be positively selected. Second, the ability to form biomolecular condensates could minimize toxic protein aggregation and could protect de novo proteins from being purged by negative selection.

To understand if de novo proteins can indeed be a source of evolutionary novelty, we analyzed their distribution in the protein sequence space relative to that of conserved and random proteins, using the protein language model ESM2-650 M. Our analysis shows that de novo proteins have unique sequence characteristics that distinguish them more clearly from conserved proteins than from random proteins, as hypothesized before (Bornberg-Bauer et al. 2021). Nevertheless, some de novo proteins indeed had a closely located conserved protein in the sequence space (Fig. 4). Together with our Foldseek analysis, this observation indicates an inherent capacity of amino acid sequences to adopt structures, and that a broad spectrum of sequence space is capable of evolving into foldable proteins (Tretyachenko et al. 2017; Heames et al. 2023; Aubel et al. 2024).

Our analysis is based on computational tools, which are always prone to some level of erroneous predictions. Furthermore, many of the deep learning-based tools have not been trained on de novo proteins and can possibly make biased predictions (Middendorf and Eicholt 2024). Therefore, our study may not provide exact and perfect answers to the different open questions about de novo proteins. All computational predictions need experimental validation. Experimental studies, especially on de novo proteins are bottlenecked by serendipity, and labor intensive techniques that are not fully optimized for proteins with such an unusual biochemistry (Eicholt et al. 2022). However, our exhaustive approach can help guide focused experimental studies, and can reduce the need for trial and error, and accidental discoveries. For example, the candidate de novo proteins with a possible structure, a specific “Molecular Function” (like hydrolysis, or RNA binding), and a propensity to form condensates, can be experimentally probed for these specific properties. Our sequence space analysis can also identify de novo proteins that are likely to adopt more conserved-protein-like properties, as a consequence of evolution. Overall, our study not only broadens our understanding of the dynamic nature of protein evolution but also serves as a guidebook for future experimental studies.

## Materials and Methods

### Dataset Curation

We used the sequence datasets from our previous study (Middendorf and Eicholt 2024). Specifically, we first obtained 6,716 orphan protein sequences from the *Drosophila* clade, and their corresponding evolutionary age, from Heames et al. (2020). From this dataset, we discarded sequences that were annotated with the same FlyBase ID. Next, we extracted the sequences whose emergence origin was annotated as “denovo” (intergenic de novo protein) or “denovo-intron” (intronic de novo protein) by Heames et al. (2020), for further analysis. Out of the 2,510 proteins sequences thus obtained, 1,481 were annotated as “denovo,” while 1,029 were described as “denovo-intron.” Based on their date of emergence, the de novo proteins were classified as young (<5 mya), intermediate (5–30 mya), and old (>30 mya) proteins (Heames et al. 2020; Middendorf and Eicholt 2024). In our filtered dataset, the three age groups consisted of 2,205, 110, and 195 proteins, respectively.

We generated 2,507 random sequences with the same distributions of amino acid composition and sequence length, as the 2,510 de novo sequences set, using a technique used in previous studies (Heames et al. 2023; Middendorf and Eicholt 2024). We generated a set of conserved protein sequences with the same sequence length distribution as the de novo proteins, by randomly sampling protein sequences from the combined proteome of 12 *Drosophila* species. After removing sequences that were duplicated or were redundant with our set of de novo proteins, we obtained a set of 2,235 unique conserved proteins. Additionally, we generated a set of randomized sequences based on the length and amino acid distribution of the selected conserved proteins.

We performed structure predictions using AF2 (v2.1.1, Jumper et al. 2021) on the High Performance Computing Cluster, PALMA II (University of Muenster). We used the predictions with the highest mean pLDDT for further analysis. We downloaded AF2-based structure predictions of conserved *Drosophila* proteins from the AlphaFold Protein Structure database (Varadi et al. 2021) for our initial analyses.

### Molecular Dynamics Simulations to Refine Structure Predictions

To analyze the stability of the predicted structures of de novo proteins, we performed molecular dynamics (MD) simulations using a previously described method (Ferruz et al. 2022), with minor modifications. We only simulated protein structures with (i) less than 30% disorder predicted by flDPnn (Katuwawala et al. 2021) and (ii) less than 95% of their residues predicted as α-helices by DSSP (Kabsch and Sander 1983) (1,468 unique proteins). We constructed the MD model and performed the simulations using the HTMD python package (Doerr et al. 2016). The model systems were built to form solvated all-atom cubic boxes. We centered our proteins at the origin of the simulation box coordinates. We used water as the solvent and added NaCl ions to neutralize the system. We used the AMBER 14SB force field (Maier et al. 2015) for all simulations. We minimized and equilibrated for 1 ns. We simulated each system for 100 ns, using the ACEMD engine (Harvey et al. 2009) with the default settings in triplicates. We evaluated the simulations with the HTMD (Doerr et al. 2016) and MDAnalysis (Michaud-Agrawal et al. 2011) python packages. We calculated the average RMSD value per trajectory for every replicate simulation for a protein, and in turn calculated a single averaged value from three replicates.

### Identifying Similar Protein Structures Using Foldseek

We searched the AlphaFold Protein Structure database (Varadi et al. 2021) clustered at 50% sequence identity (AFDB50), for structures similar to the predicted structures of our de novo, random, and conserved proteins, using Foldseek (v.8.ef4e960, van Kempen et al. 2024). We applied the same filtering criteria our query proteins that we used for the MD simulations. For de novo proteins, we used the protein structures refined after 100 ns of MD simulation. We downloaded precomputed AFDB50 database via Foldseek’s database module. We searched for similar structures using the “easy-search” module of Foldseek with the default settings. We did not filter the results or queries based on the pLDDT values. We discarded all hits to proteins within the *Drosophila* clade, to exclude hits to orthologous de novo proteins.

To identify and annotate potential known protein structural domains in the de novo proteins, we searched the protein data bank database (PDB, January 2024, Berman et al. 2000) for structures that were similar to that of de novo proteins (MD-refined). We used Foldseek for this analysis with the same settings as we did before for AFDB50. We discarded hits with a TM-score less than 0.5 (Zhang and Xu 2010). We retrieved the annotated ECOD domains of the highest scoring hits, from the ECOD database (Release: 20230309, Cheng et al. 2014) if the structural alignment of the de novo protein covered at least 80% of the target structure from the PDB. In all cases, we only used the highest TM scoring hit out of the three MD replicates for further analysis. We additionally performed hmmscan (standard settings) (Finn et al. 2011) locally against ECOD database (Release: 20230309, Cheng et al. 2014) and Pfam (v. 37.0, Mistry et al. 2021) on the 42 de novo sequences found to have structural similarity to ECOD architectures. We manually checked the T-level of the aligned target domains in the ECOD database.

### Predicting Protein Function Using DeepFri

To understand the potential function of de novo, and random proteins, we predicted their gene ontology (GO) terms using DeepFRI (Gligorijević et al. 2021). We used their AF2 predicted 3D-structures (MD refined for de novo proteins) as input and identified hits with a score ≥ 0.5. We summarized the predicted GO terms to a small list of terms using REVIGO (Supek et al. 2011), and measured semantic similarity using SimRel (Sæbø et al. 2015). We visually, identified clusters within the semantic space and annotated them with a term that summarizes the GO terms within them.

### Analysis of De Novo Proteins That Form Biomolecular Condensates

We predicted the potential of de novo proteins to form biomolecular condensates, using PICNIC (Hadarovich et al. 2023). Because PICNIC makes predictions based on metrics derived from AF2 and IUPred2A predictions, we applied further filtering steps of the results in order to obtain a set of high-confidence condensate-forming de novo proteins. To this end, we retrieved all the proteins from the CD-CODE database (Rostam et al. 2023), that were experimentally shown from biomolecular condensates in cellulo or in vivo. This set of 175 proteins served as our positive control. Next, we retrieved sequence features associated with the biological functions of intrinsically disordered regions of proteins (Zarin et al. 2021), using the scripts provided in the idr.mol.feats GitHub repository. We discarded the specific features—*aromatic_spacing*, omega_aromatic*, and kappa*, and features that count the appearance of specific binding motifs. We normalized all the features that are directly influenced by the sequence length (e.g. amino acid counts), to the sequence length of the corresponding proteins. We subsequently clustered the sequences based on the computed features using hdbscan (McInnes et al. 2017) with a minimal cluster size of 100 the *min_samples* parameter set to a value of 50. We considered a de novo protein to be a high-confidence condensate-forming protein, if it shared a cluster with a large fraction of proteins from the CD-CODE database, and had a PICNIC score ≥ 0.5. We used both original AF2 predicted structures and MD refined structures for our analysis and found that MD refinement only weakly affects predictions by PICNIC (supplementary Fig. S7, Supplementary Material online).

### Mapping Protein Sequences to a Numerical Space Using Protein Language Model

To understand how de novo and random protein sequences are located within the protein sequence space relative to conserved proteins, we used the protein language model ESM2 with 650 million parameters (ESM2-650 M) (Lin et al. 2023). Specifically, we used the language model to convert each sequence to a numerical vector with 1,280 elements. More specifically, ESM2-650 M assigns each amino acid residue in a protein sequence, a 1,280D vector of “embeddings.” For each protein we calculated the multivariate mean of the embedding vectors from every amino acid residue. We calculated the Manhattan distance (or L1 norm) between the numerical sequences of every pair of proteins in our combined dataset of de novo, random and conserved proteins. We applied Mann–Whitney test to the pairwise distances to analyze if proteins of one class (e.g. de novo) are farther from that of another class (e.g. conserved), than with each other. For proteins of one class, we also used the pairwise distances to identify the nearest neighboring protein from the other class.

### Data and Statistical Analysis

Data were analyzed with Python (v3.9.18) (Van Rossum and Drake 2009), using the following packages: Pandas (v1.5.3) (Reback et al. 2022), NumPy (v1.26) (Harris et al. 2020), SciPy (v1.11.3) (Virtanen et al. 2020), Seaborn (v.0.12.2) (Waskom 2021), and BioPython (v1.80) (Cock et al. 2009). We generated the plots using Matplolib (v3.4.3) (Hunter 2007). We performed the Pearson’s $\chi^{2}$-tests and Fisher’s Exact test using “chisquare” or “fisher_exact” from the scipy.stats package. We analyzed protein sequence space with Julia programming language using the packages Distances.jl (v0.10.11) and HypothesisTests.jl (v 0.11.0).

## Supplementary Material

evae176_Supplementary_Data

## Data Availability

Datasets are publicly available on Zenodo. All scripts are freely available on GitHub: *https://github.com/LasseMiddendorf/SequenceAndFunctionalSpaceOfDrosophilaDeNovoProteins*.
