# Supplementary material for: Sequence, Structure, and Functional Space of Drosophila De Novo Proteins
Source: Genome Biol Evol. 2024 Aug 30;16(8):evae176. doi: 10.1093/gbe/evae176 (PMC11363682; doi:10.1093/gbe/evae176)
Supplement: evae176_Supplementary_Data [file evae176_supplementary_data.zip › supplementary_figures/supplementary_figures/Supp_figures_legends.rtf]

\textbf{Figure S1: Structural diversity of \textit{de novo} proteins, before MD refinement.} The predicted protein structures of randomly generated sequences (\textbf{A}), \textit{de novo} protein (\textbf{B}), and conserved proteins (\textbf{C}) were queried against the AlphaFold database (AFDB50) excluding proteins from \textit{Drosophila}. Only proteins with less than 30\% of their residues being predicted to be disordered and less than 95\% with a DSSP annotation of being \textalpha{}-helical were considered for the analysis. Shown is the distribution of the highest TM-score found for each protein in the three datasets. (\textbf{D}) Overview of the structural classes and ECOD architectures of \textit{de novo} proteins. The protein class (inner circle) was assigned to all \textit{de novo} proteins queried against the AFDB50 based on the DSSP annotations of the predicted protein structures. Proteins containing no residues annotated as \textalpha{}-helices or \textbeta{}-sheets were classified as \textit{all b} or \textit{all a}, respectively. Protein structures containing residues annotated as \textalpha{}-helices and \textbeta{}-sheets were classified as \textit{Mixed}. For the annotation of ECOD architectures in the predicted structures of \textit{de novo} protein, the structures were queried against the PDB and assigned with the ECOD domain of the highest ranking hit if the alignment covered at least 80\% of the target structure.\\\\\textbf{Figure S2: Relationship of average RMSD of MD trajectories and TM-score to closest hit in AFDB50.} We analyzed the relationship between the average RMSD of the MD trajectories of \textit{de novo} proteins and the TM-score of their highest-scoring structural alignment in AFDB50. We find a weak correlation between the two values (Pearson $\rho = -0.31$, $P = 1.82\times 10^{-29}$), but only a low fraction of the variance is explained by them ($R^2 = 0.01$).\\\\\textbf{Figure S3: Count of occurrence of age groups in 42 \textit{de novo} proteins found to be structurally similar to ECOD architectures} Counting the occurrences of age groups defined by time of emergence described in Heames \textit{et al.} (2020) (young: <5 mya, intermediate: 5-30 mya, old: >30 mya), we find that the majority are young \textit{de novo} proteins. Frequencies of the three age groups in the 42 \textit{de novo} proteins with an annotated ECOD domain were as expected by the overall frequencies of the age groups (Pearson's $\chi^2$-Test; $P = 0.99$).\\\\\textbf{Figure S4: Structural similarity of high-pLDDT protein structures to AlphaFold database} Similar structures in the AlphaFold database for high-pLDDT structure predictions only TM-Score distribution of predicted protein structures of (\textbf{A}) random, (\textbf{B}) \textit{de novo}, and (\textbf{C}) conserved proteins with a pLDDT value \textgreater{}= 70 queried against the AlphaFold database (AFDB50) using Foldseek. The hit with the highest TM-score was chosen for each protein.\\\\\textbf{Figure S5: RMSD trajectories of selected \textit{de novo} proteins} Root mean square deviation (RMSD) of Droso\_1087, Droso\_1446, and Droso\_4480 over 100 ns of molecular dynamics simulations. Simulations were performed as triplicates for all proteins.\\\\\textbf{Figure S6: Robustness analysis of high-confidence condensate forming \textit{de novo} proteins.} (\textbf{A}) We analyzed the n\_neighbors hyperparameter for UMAP, adjusting it before identifying clusters with HDBSCAN. For each n\_neighbors value, we analyzed cluster composition and considered \textit{de novo} proteins with a PICNIC score \textgreater{}= 0.5 in clusters containing the majority of CD-CODE proteins as high confidence condensate forming. The number of high confidence \textit{de novo} proteins is similar for n\_neighbors \textless{}= 20, but drops to 21 for n\_neighbors = 25. (\textbf{B}) To assess robustness, we investigated how many high-confidence \textit{de novo} proteins are shared between different sets identified with varying n\_neighbors. Numbers at the intersections represent shared high-confidence proteins. All 63 identified high-confidence \textit{de novo} proteins are found with multiple n\_neighbors values.\\\\\textbf{Figure S7: Influence of using MD-refined structures for PICNIC predictions.} We predicted the condensate forming potential of \textit{de novo} proteins with PICNIC using either AF2 predictions or their MD-refined AF2 predictions. The distribution of the change in the PICNIC score when using MD-refined structures in comparison to AF2 predictions shows that using MD-refined structures only weakly affects PICNIC predictions.
