# Supplementary figures and images for: Sequence, Structure, and Functional Space of Drosophila De Novo Proteins
Source: Genome Biol Evol. 2024 Aug 30;16(8):evae176. doi: 10.1093/gbe/evae176 (PMC11363682; doi:10.1093/gbe/evae176)

A)

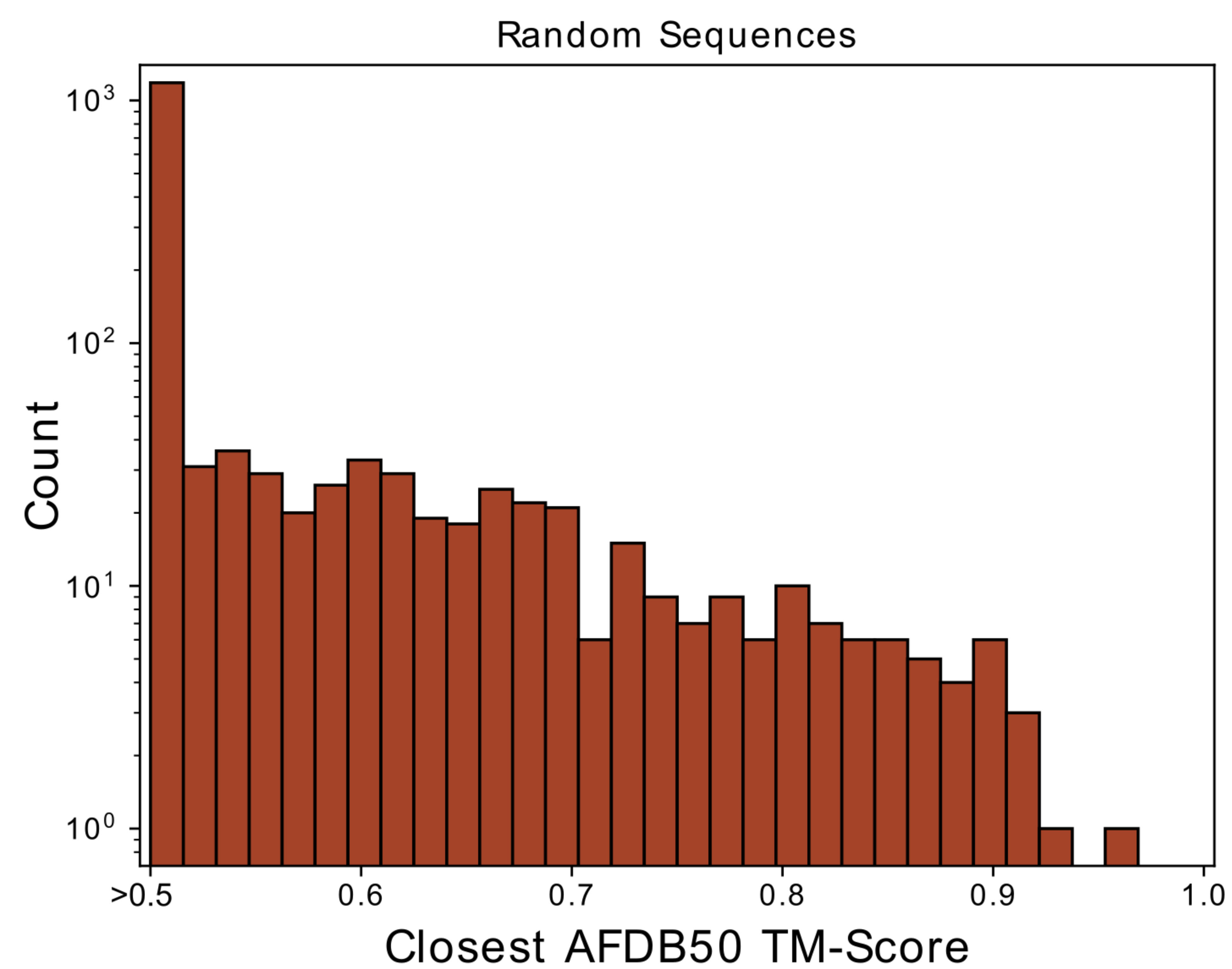

B)

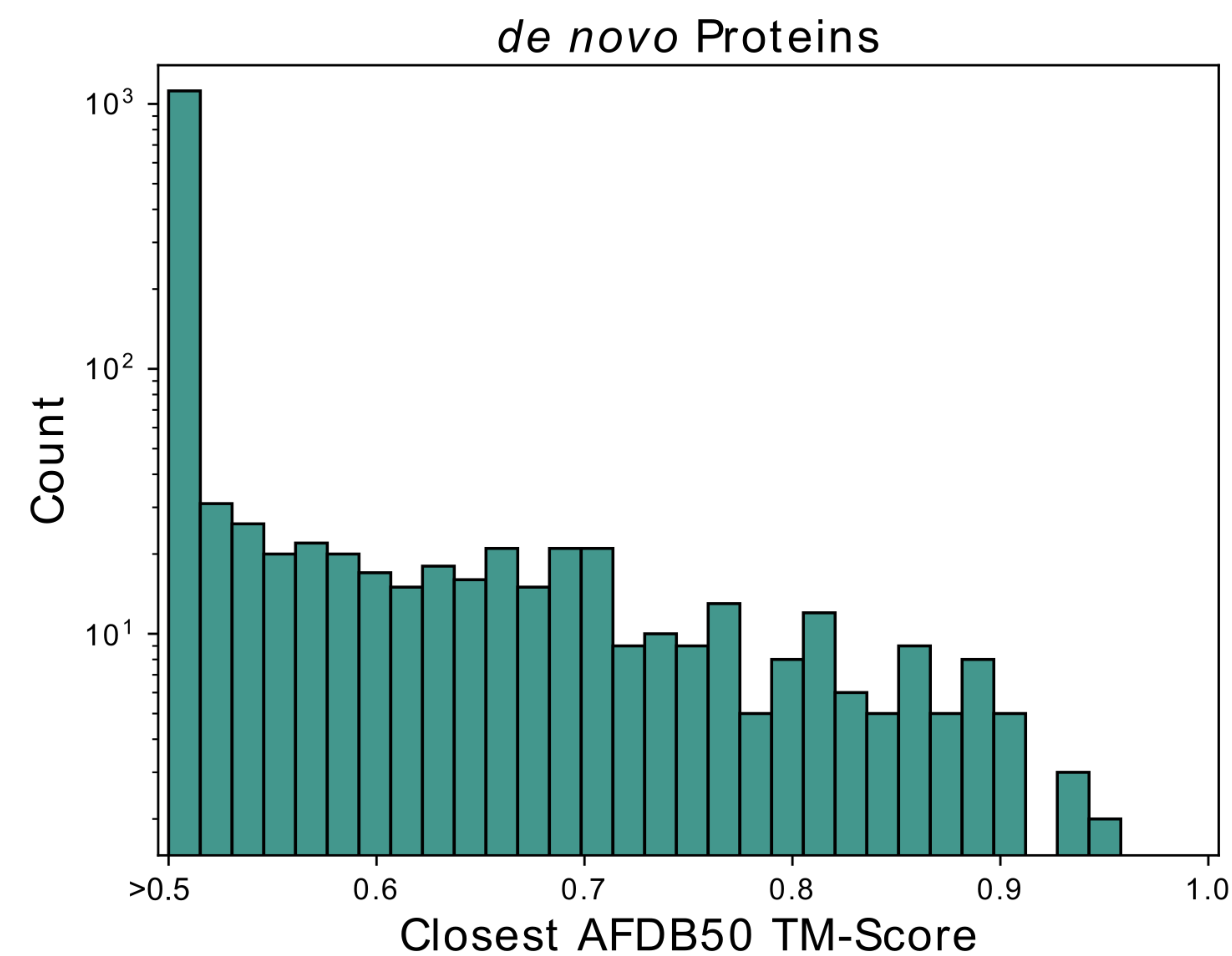

C)

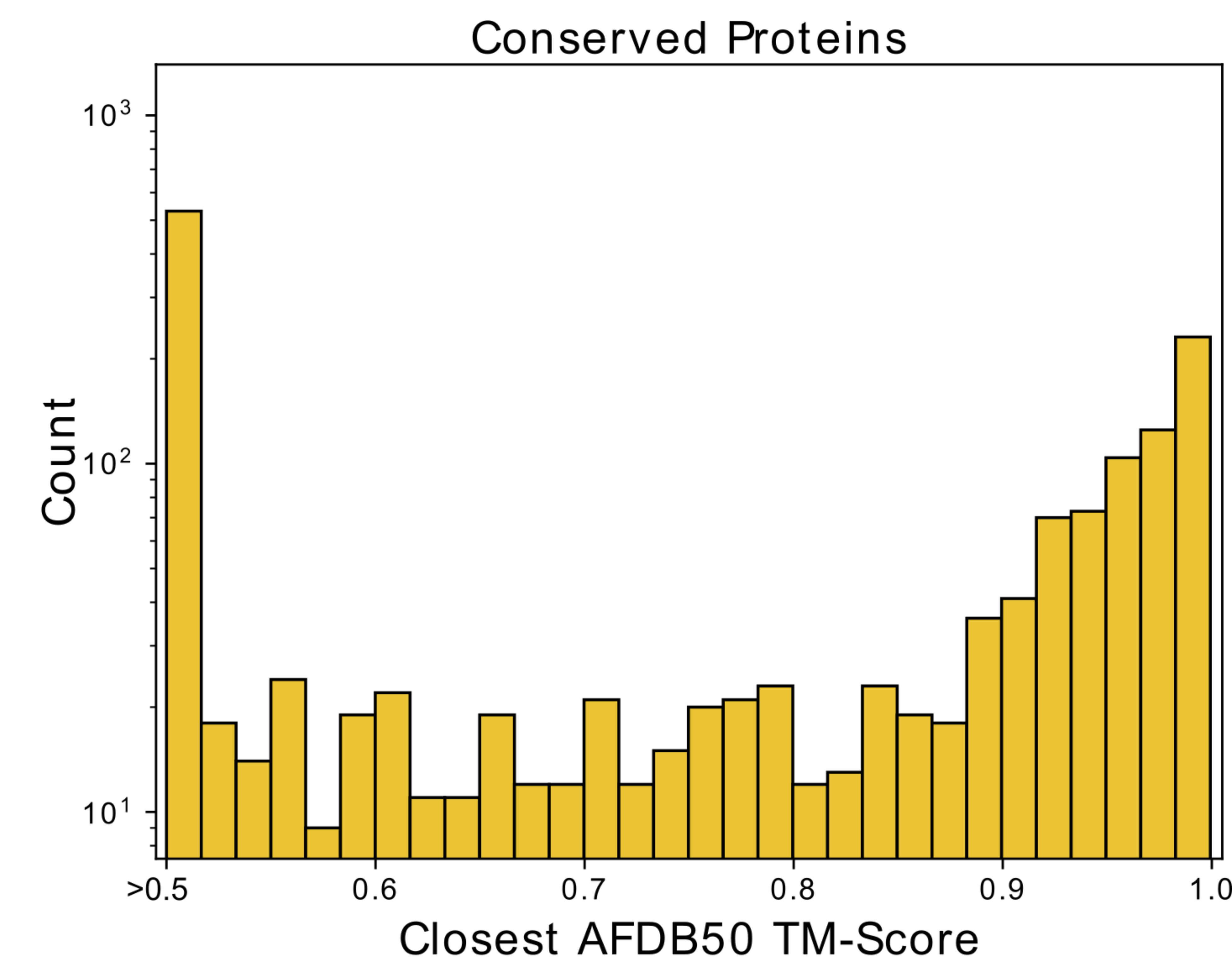

D)

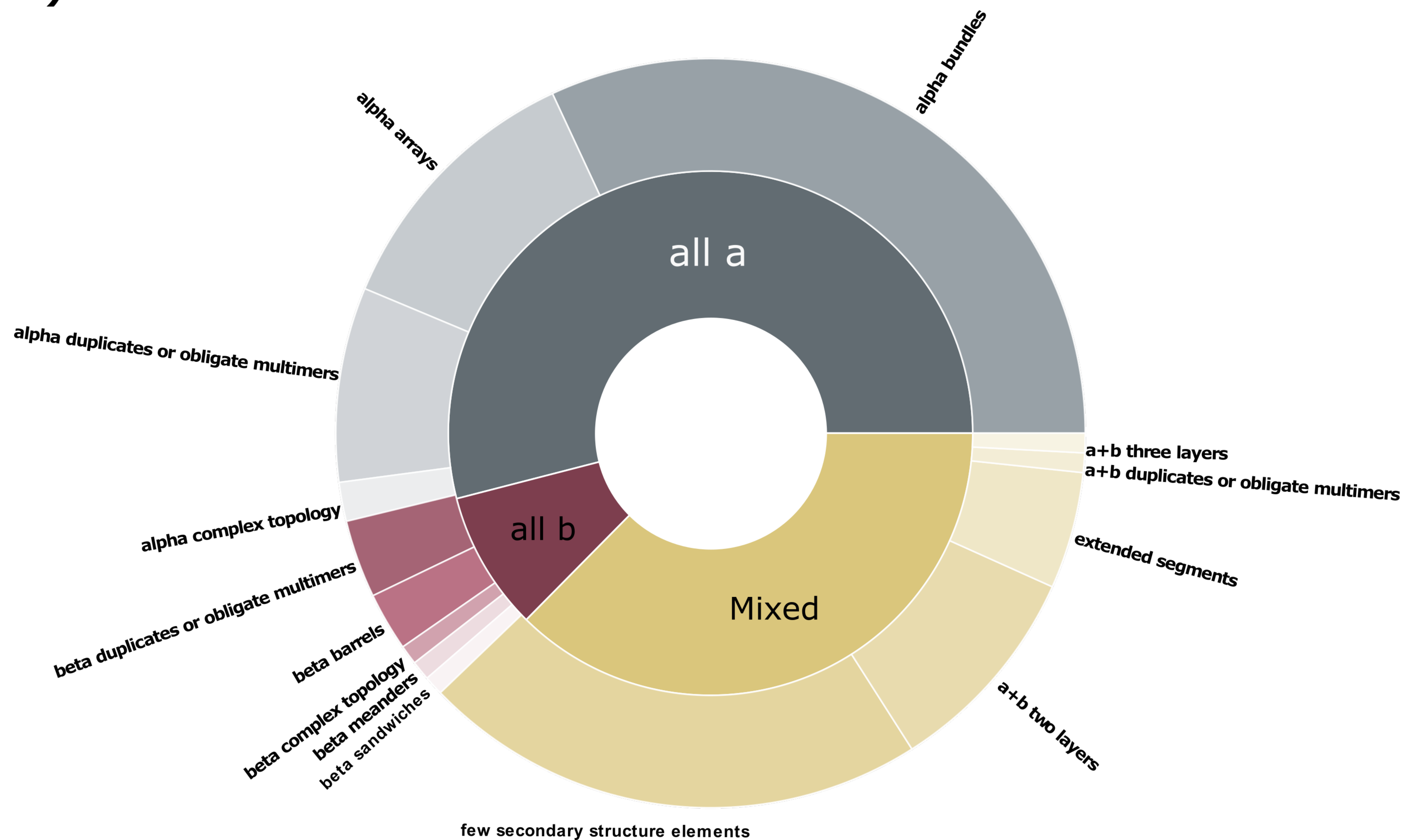

Proteins with Class Annotation: 1468

Proteins with Architecture Annotation: 119

Supplement: evae176_Supplementary_Data [file evae176_supplementary_data.zip › supplementary_figures/supplementary_figures/Figure_S1.pdf]

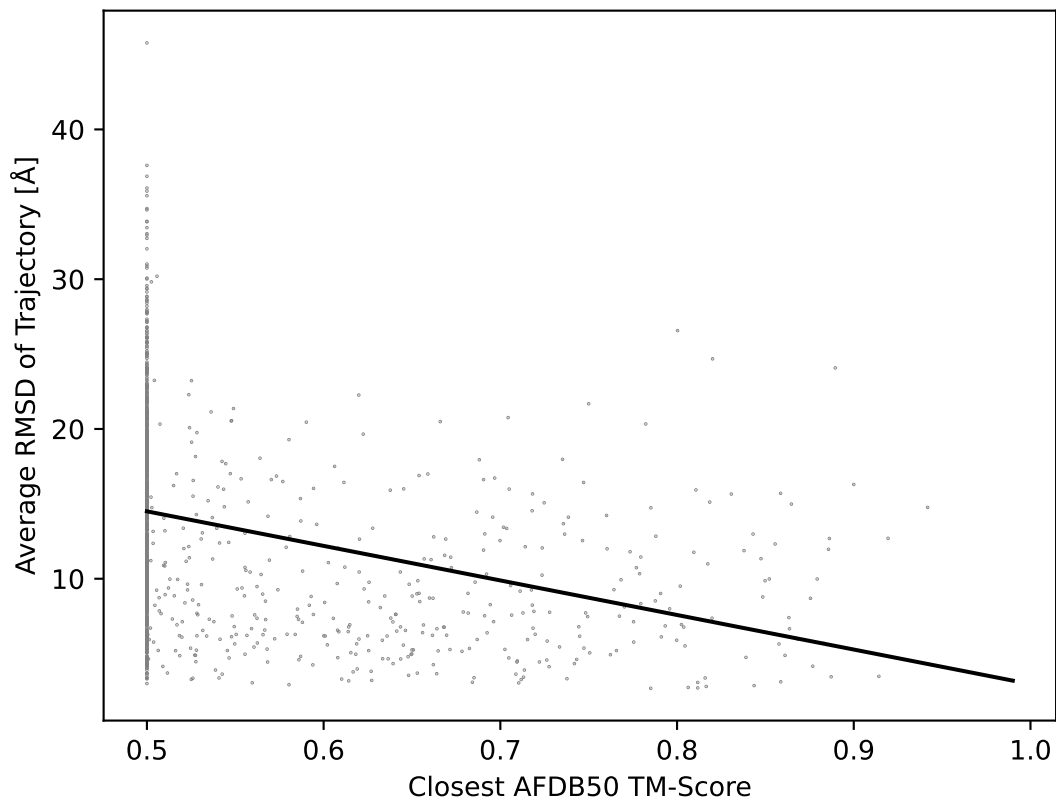

Supplement: evae176_Supplementary_Data [file evae176_supplementary_data.zip › supplementary_figures/supplementary_figures/Figure_S2.pdf]

Age Groups of de novo proteins annotated with ECOD architecture

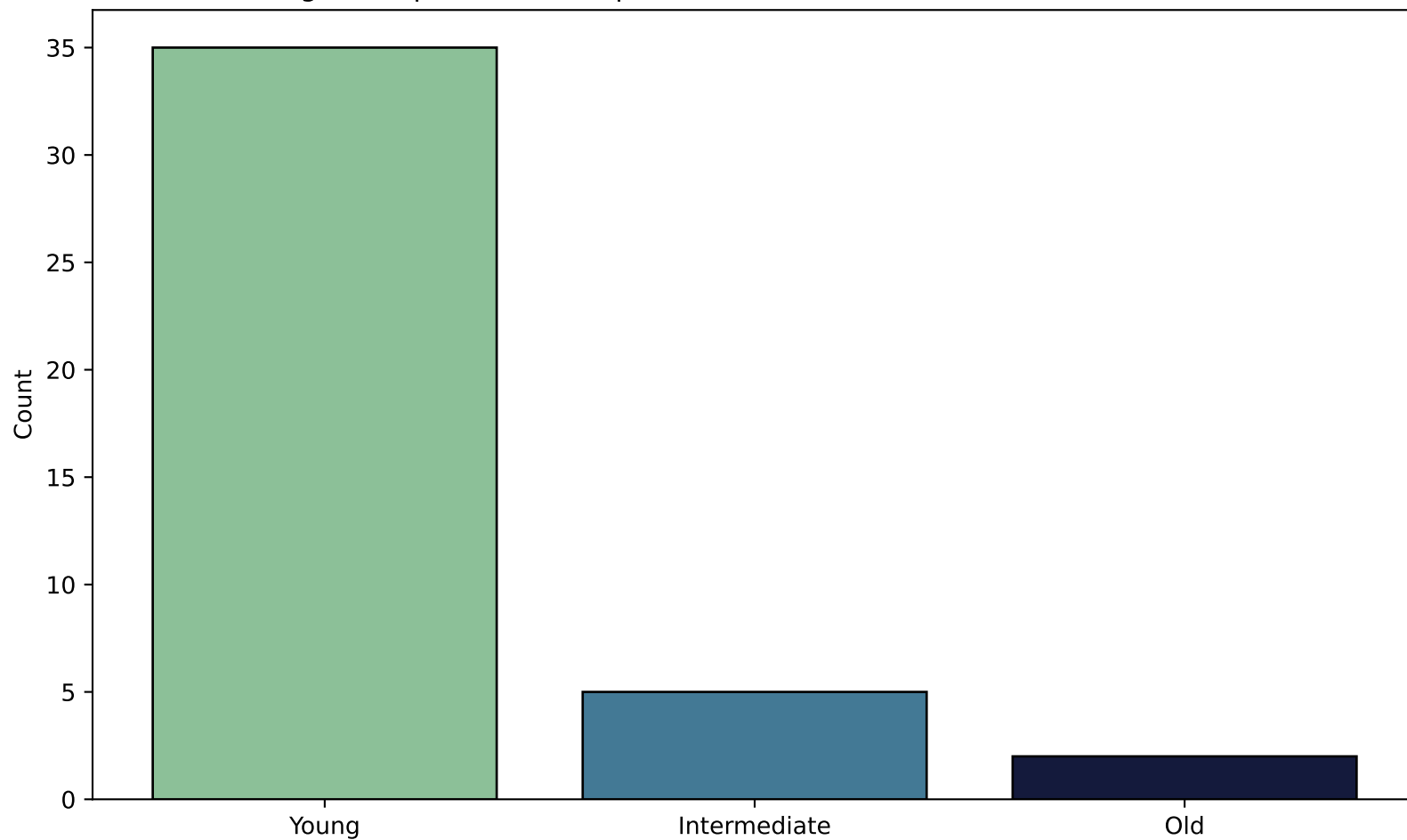

Supplement: evae176_Supplementary_Data [file evae176_supplementary_data.zip › supplementary_figures/supplementary_figures/Figure_S3.pdf]

A)

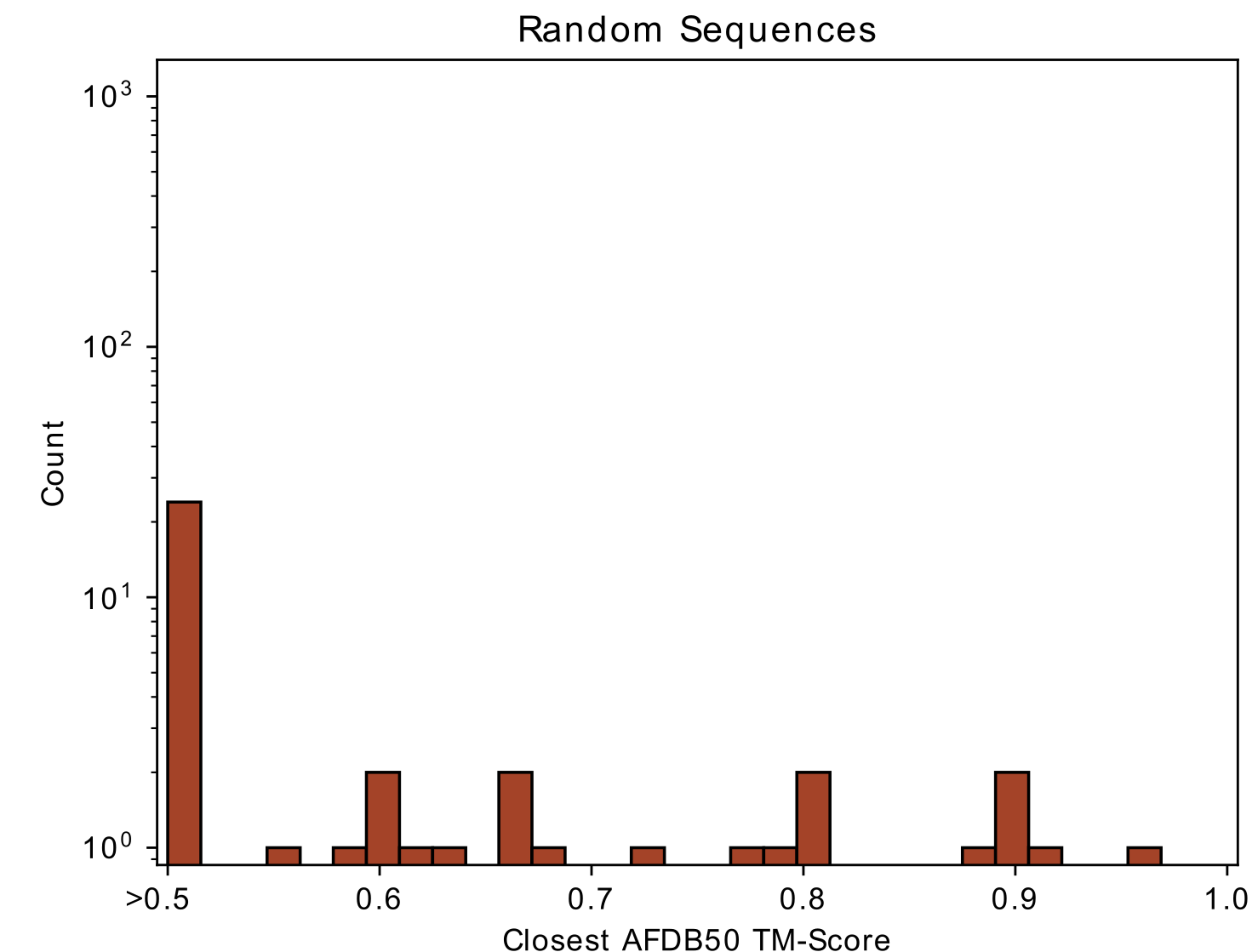

B)

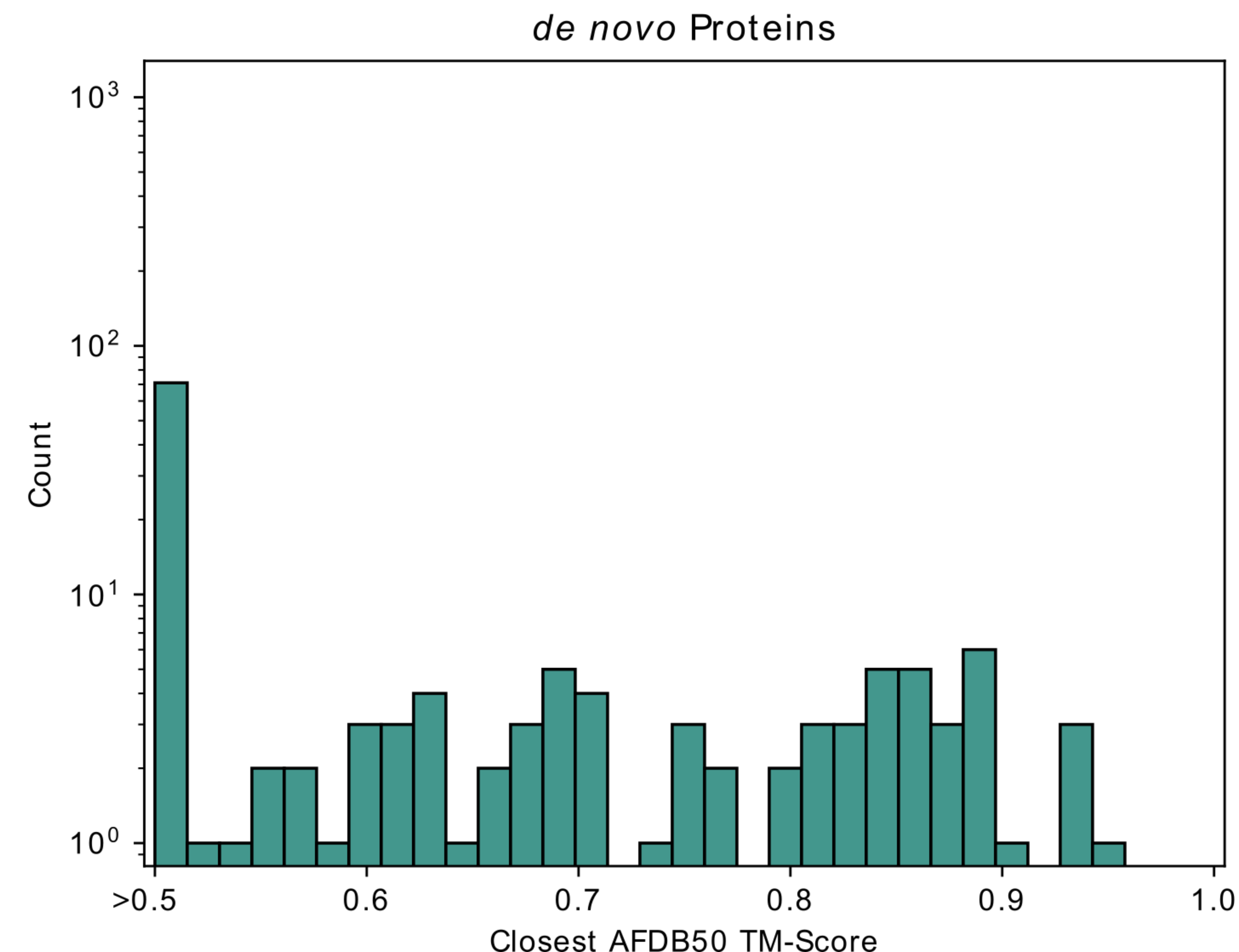

C)

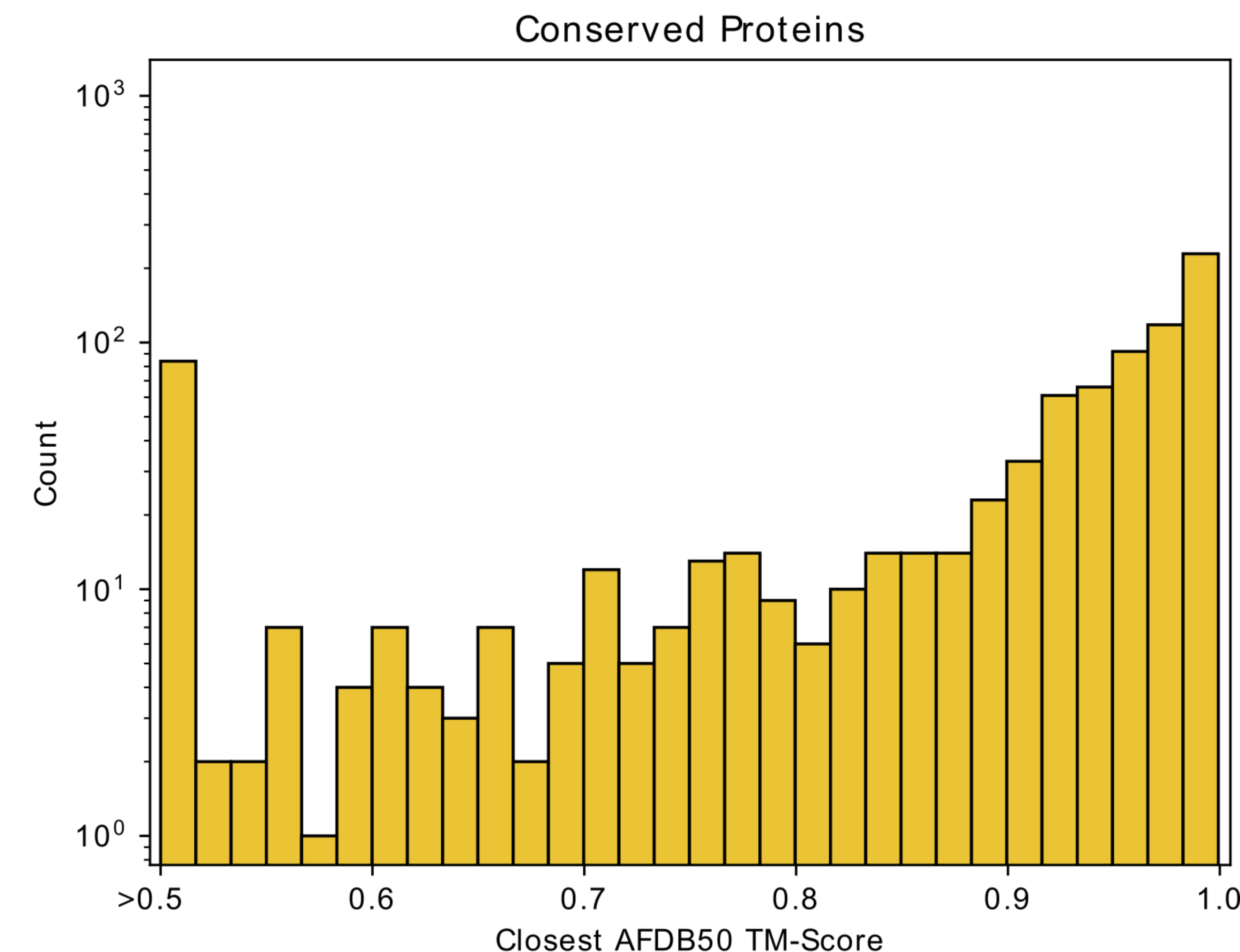

Supplement: evae176_Supplementary_Data [file evae176_supplementary_data.zip › supplementary_figures/supplementary_figures/Figure_S4.pdf]

A)

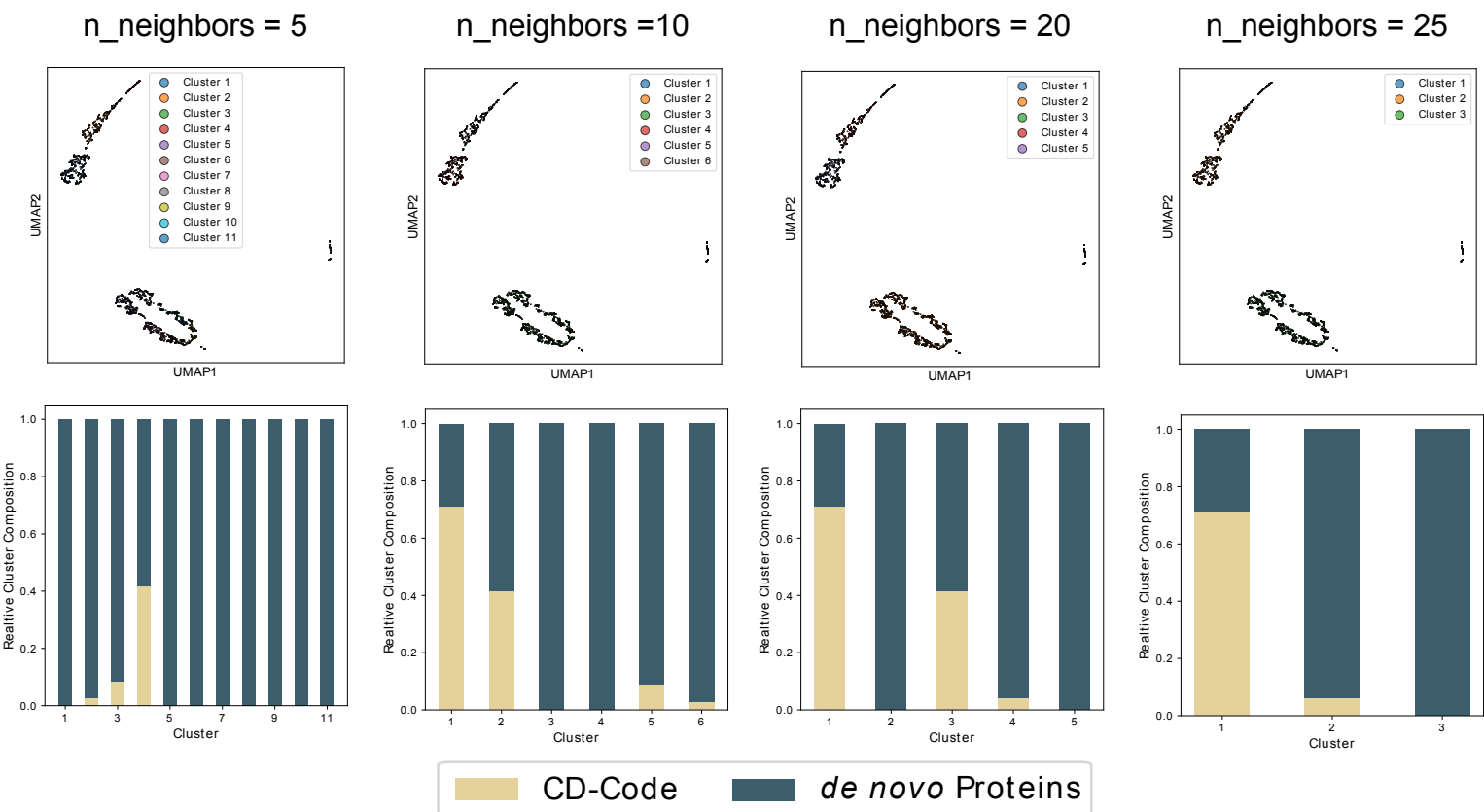

B)

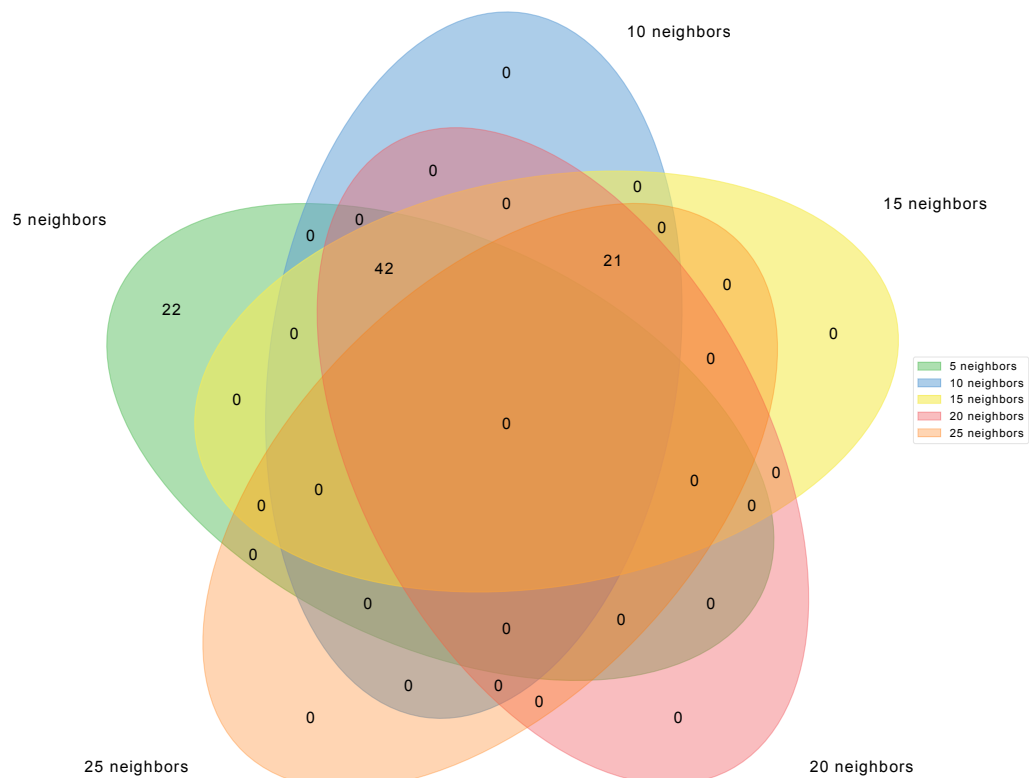

Supplement: evae176_Supplementary_Data [file evae176_supplementary_data.zip › supplementary_figures/supplementary_figures/Figure_S6.pdf]

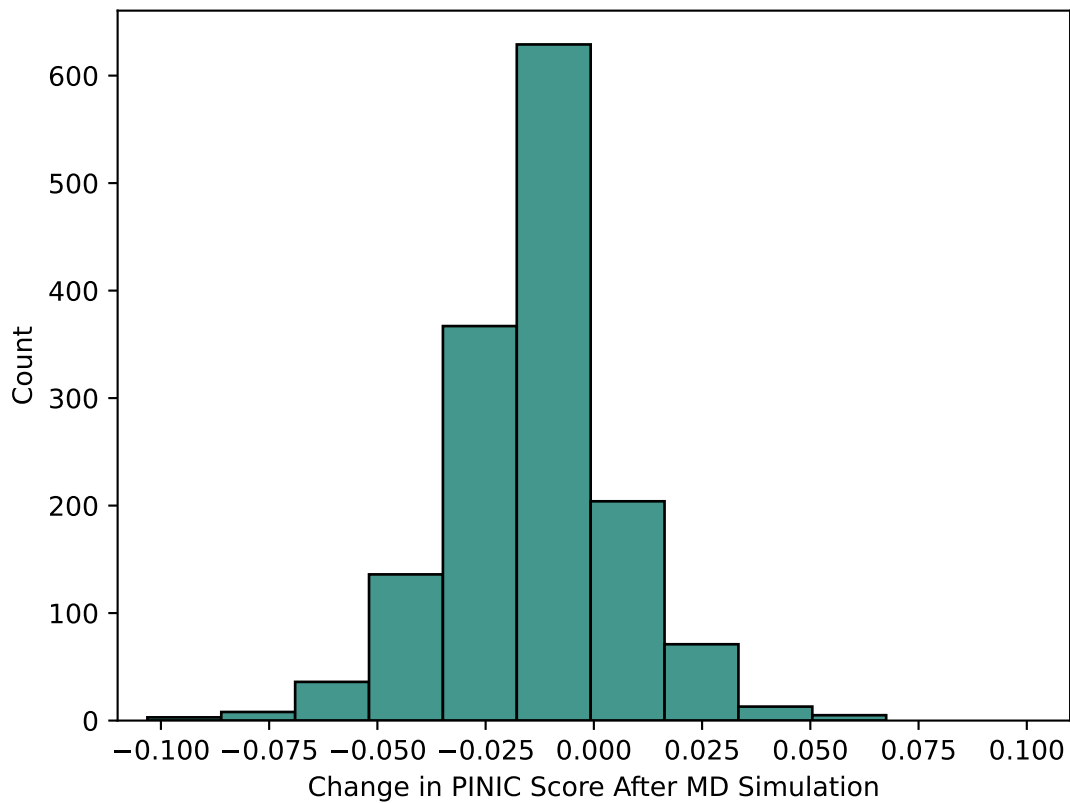

Supplement: evae176_Supplementary_Data [file evae176_supplementary_data.zip › supplementary_figures/supplementary_figures/Figure_S7.pdf]
