# Supplementary material for: Sequence, Structure, and Functional Space of Drosophila De Novo Proteins
Source: Genome Biol Evol. 2024 Aug 30;16(8):evae176. doi: 10.1093/gbe/evae176 (PMC11363682; doi:10.1093/gbe/evae176)
Supplement: evae176_Supplementary_Data [file Figure_S5.pdf › supplementary_figures/supplementary_figures/Figure_S5.pdf]

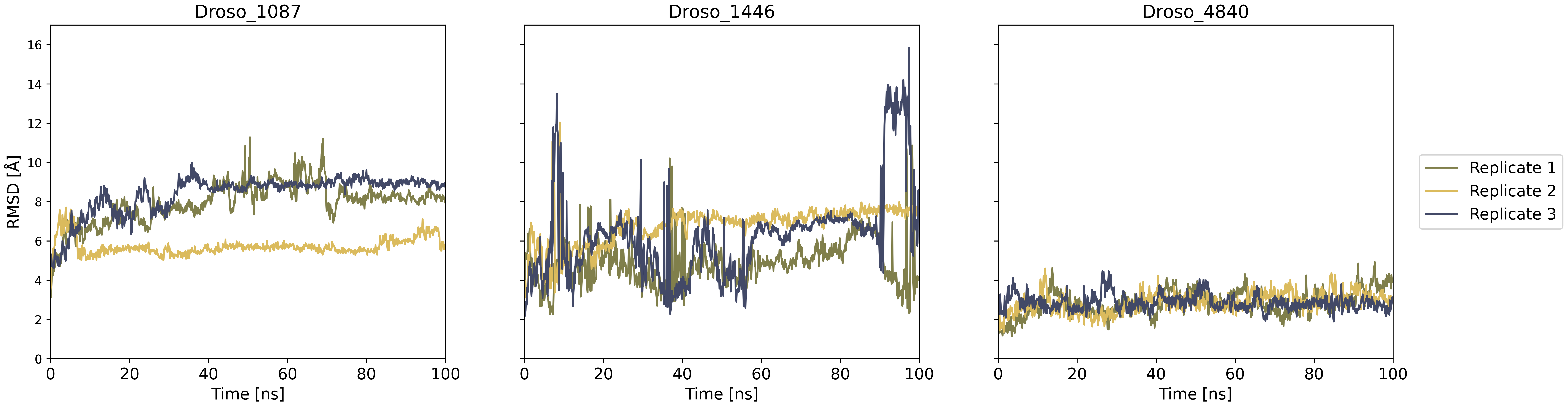
Droso_1087
Droso_1446
Droso_4840
RMSD [Å]
Time [ns]
Time [ns]
Time [ns]
0
2
4
6
8
10
12
14
16
0
20
40
60
80
100
Replicate 1
Replicate 2
Replicate 3
